# Atypical Follicular–Pigmentary Variant of Hailey–Hailey Disease: A Diagnostic Dilemma

**DOI:** 10.1002/ccr3.73022

**Published:** 2026-06-23

**Authors:** Sandesh Shah, Joshana Shrestha, Rabin Baniya, Mohan Bhusal, Krishangi Pradhan, Radhika Maharjan

**Affiliations:** ^1^ Department of Dermatology Nepal Medical College and Teaching Hospital Kathmandu Nepal; ^2^ Department of Prosthodontics B.P Koirala Institute of Health Sciences Dharan Nepal

**Keywords:** acantholytic, benign familial pemphigus, dilapidated, Hailey–Hailey disease

## Abstract

Hailey–Hailey disease (HHD) is a rare autosomal dominant acantholytic dermatosis characterized by recurrent erosive plaques in intertriginous areas. Atypical presentations can resemble other acantholytic disorders, making diagnosis challenging. A 38‐year‐old male presented with recurrent pruritic lesions over flexural regions, worsened by heat and sweating. Although classical erosive plaques were noted over the neck and axillae, lesions over the groin and other flexures showed a predominant papular and follicular morphology with marked post‐inflammatory pigmentation, mimicking other acantholytic dermatoses. Histopathology revealed suprabasal acantholysis with a “dilapidated brick wall” pattern. Clinicopathological correlation established the diagnosis of HHD. Atypical papular forms of HHD may mimic other acantholytic dermatoses, complicating diagnosis. Recognition of such morphological variation and clinicopathological correlation is essential for accurate diagnosis.

## Key Clinical Message

Hailey–Hailey disease typically presents with erosive plaques in flexural areas; however, atypical morphological variants may occur and resemble other acantholytic dermatoses, where clinicopathological correlation aids in accurate diagnosis.

## 1 Introduction

Hailey–Hailey disease (HHD), also known as benign familial pemphigus, is a rare autosomal dominant acantholytic disorder caused by mutations in the *ATP2C1* gene, which impair keratinocyte adhesion and epidermal integrity [1]. It usually presents in the second to fourth decades of life, with recurrent vesicles, erosions, fissures, crusting, and macerated plaques, predominantly involving intertriginous areas such as the neck, axillae, groin, and inframammary folds [1, 2]. Exacerbations are commonly triggered by heat, sweating, friction, and secondary infection, contributing to a chronic relapsing course [1, 2].

Despite its characteristic flexural distribution, HHD can pose a diagnostic challenge due to clinical and histopathological overlap with other acantholytic dermatoses, particularly Darier's disease, Grover's disease (also known as transient acantholytic dermatosis), and Dowling‐Degos disease [3, 4, 5]. Although histopathology typically shows suprabasal acantholysis with a characteristic “dilapidated brick wall” appearance, clinicopathological correlation is essential when the clinical morphology is atypical [1, 6].

We report a case of a 38‐year‐old male with recurrent flexural dermatosis showing atypical papular and follicular morphology with prominent post‐inflammatory pigmentation, in whom differentiation from other acantholytic dermatosis posed a diagnostic challenge.

## 2 Case History/Examination

A 38‐year‐old male presented with complaints of reddish to brownish, elevated lesions over the neck, bilateral axillae, groin, lower abdominal folds, popliteal fossae, and olecranon fossae for 4 months. The lesions were associated with itching and occasional burning sensations.

The patient gave a history of similar lesions 1 year prior, during the summer, that were aggravated by sweating and friction but were then limited to the neck and axillae. There was no family history of a similar illness. The patient had no known systemic illness and no history of drug intake.

**FIGURE 1:**
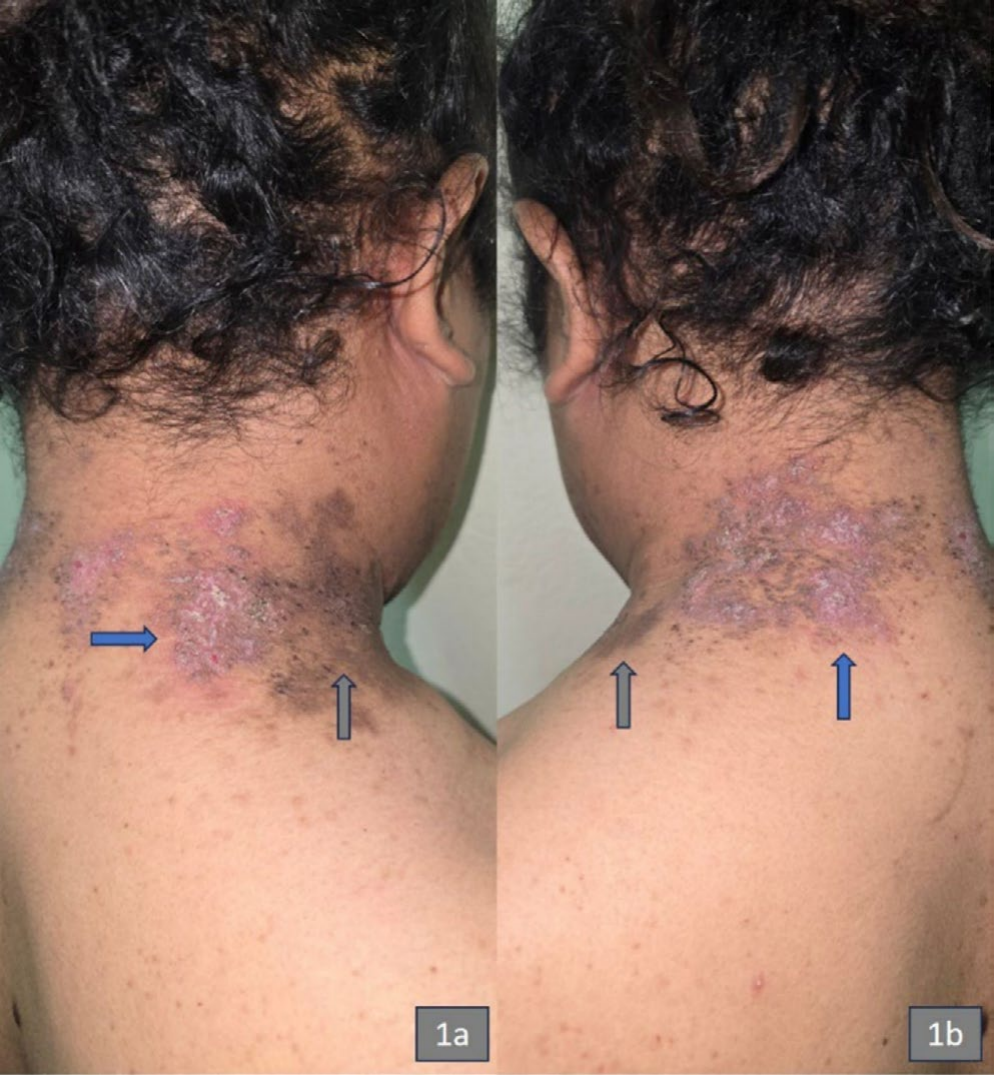
(a, b) Blue arrows indicate active erythematous plaques with scaling, crusting, and superficial erosions over the nape of the neck, whereas gray arrows indicate areas of post‐inflammatory hyperpigmentation.

**FIGURE 2:**
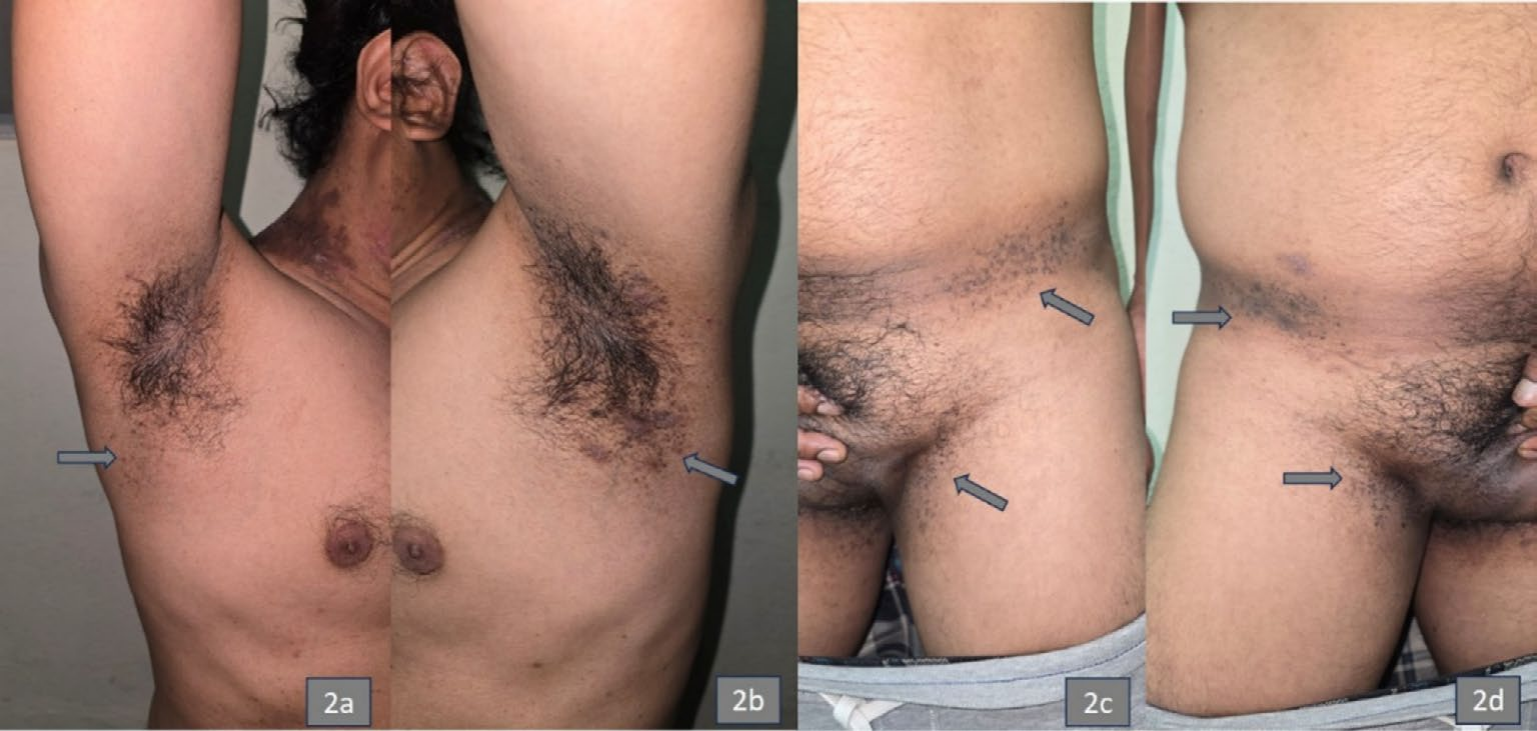
(a–d) Gray arrows indicate lesions over the bilateral axillae, bilateral lower abdominal folds, and bilateral groins, showing hyperpigmented plaques with papular and follicular morphology and prominent post‐inflammatory pigmentation.

**FIGURE 3:**
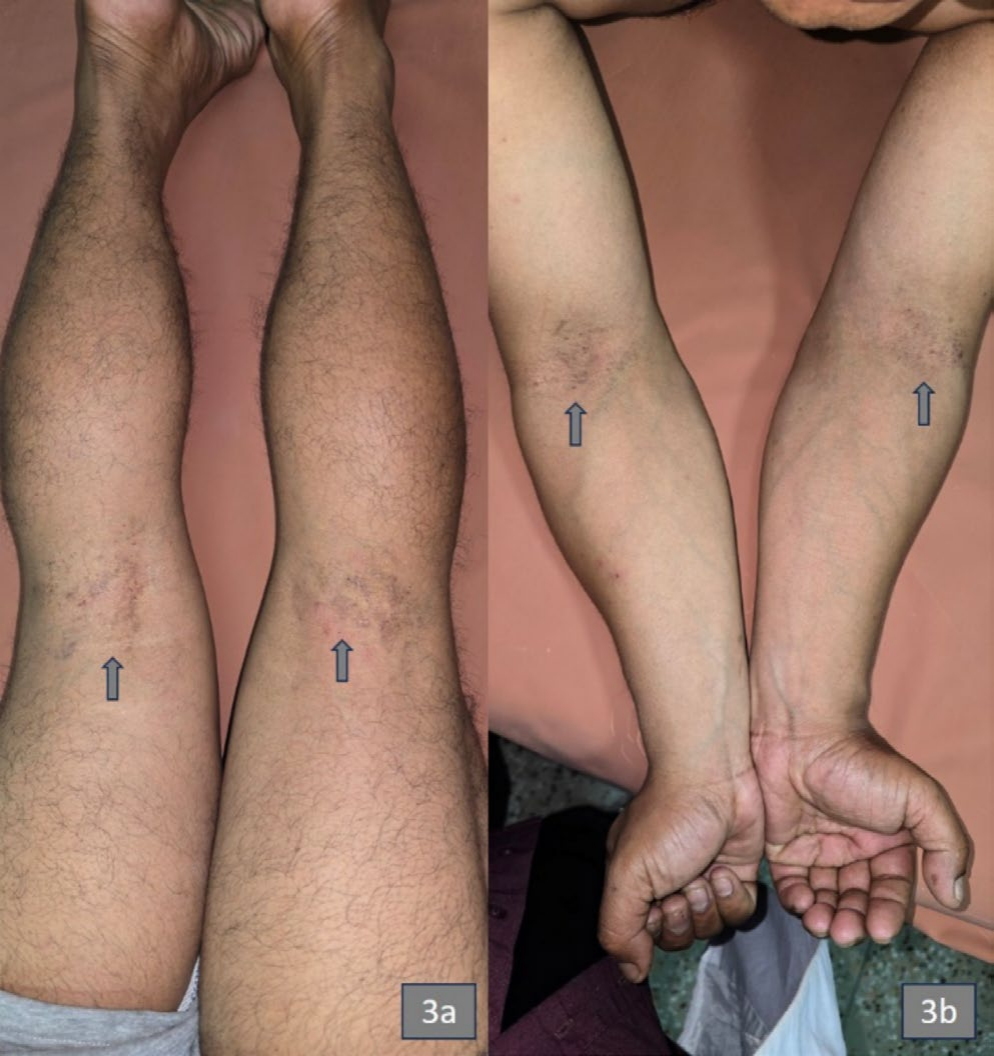
(a, b) Gray arrows indicate lesions over flexural areas of upper and lower limbs showing predominantly papular morphology with background post‐inflammatory pigmentation.

Cutaneous examination revealed multiple hyperpigmented to erythematous plaques predominantly involving intertriginous and flexural areas, including the nape of the neck (Figure 1), bilateral axillae, groin, lower abdominal folds (Figure 2), and flexural aspects of the limbs (Figure 3). The lesions were variably well‐ to ill‐defined, with surface scaling, crusting, and areas of superficial erosion. Areas of maceration and excoriation were noted, suggestive of chronicity with repeated frictional aggravation.

In addition, lesions over the bilateral groins, lower abdominal folds, and flexural aspects of the limbs demonstrated multiple pinpoint to 2–3 mm hyperpigmented macules and papules coalescing into plaques, some with keratotic changes and reticulate to patchy pigmentation (Figures 2 and 3). These areas exhibited a predominantly papular and follicular morphology with prominent post‐inflammatory hyperpigmentation, in contrast to the more erosive and crusted plaques over the neck and axillae.

No mucosal involvement, nail changes, or systemic abnormalities were observed.

## 3 Diagnosis and Treatment

Based on the clinical presentation, a differential diagnosis of Hailey–Hailey disease, Darier's disease, transient acantholytic dermatosis (Grover's disease), and Dowling‐Degos disease was considered.

**FIGURE 4:**
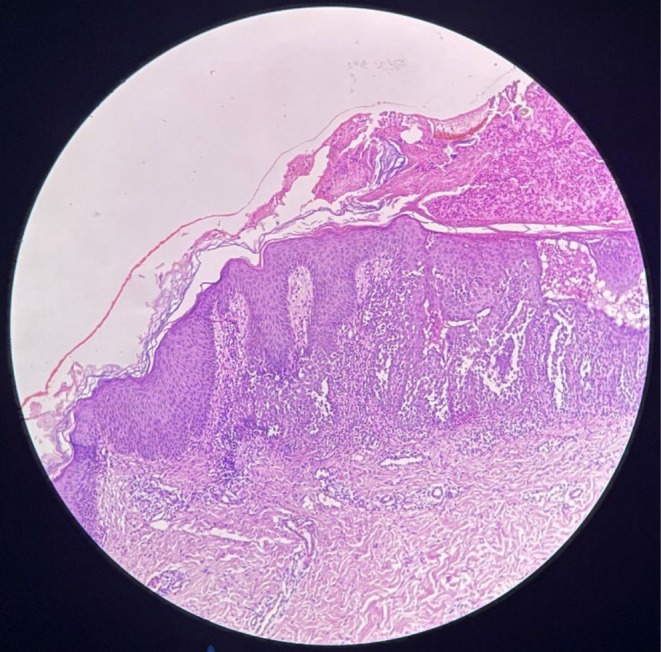
Hematoxylin and eosin stain (10×) showing epidermal changes with suprabasal cleft formation and acantholysis, along with overlying crusting and underlying dermal inflammatory infiltrate.

**FIGURE 5:**
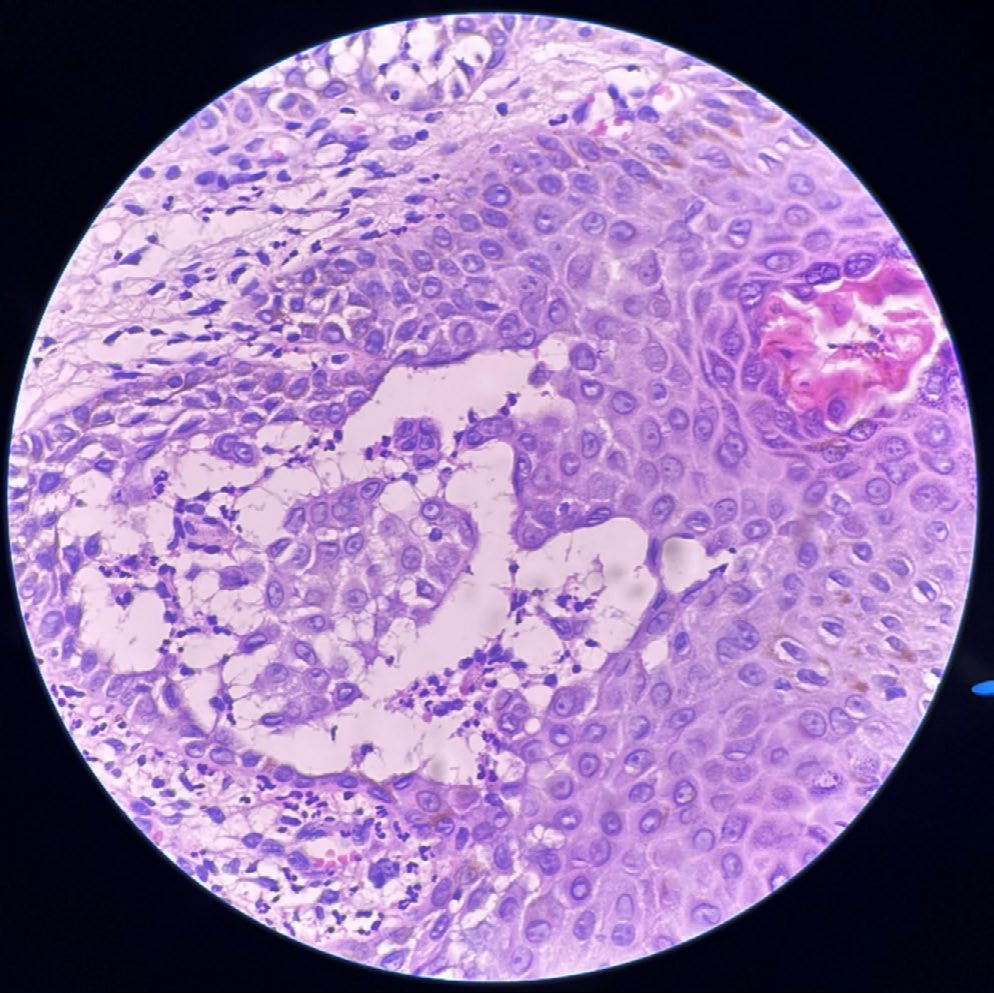
Hematoxylin and eosin stain (40×) demonstrating suprabasal acantholysis with separation of keratinocytes, producing the characteristic “dilapidated brick‐wall” appearance.

A punch biopsy was obtained from a lesion on the nape of the neck. Histopathological examination showed an epidermis lined by keratinized stratified squamous epithelium, with areas of suprabasal acantholysis and separation of keratinocytes (Figure 4), imparting the classical ‘dilapidated brick wall’ appearance characteristic of Hailey–Hailey disease (Figure 5). There was thinning of the epidermis with flattening of the rete ridges. The underlying dermis showed edema and a chronic inflammatory infiltrate, with pigment incontinence. No evidence of atypia or malignancy was observed. These findings, in conjunction with clinical features, were consistent with Hailey–Hailey disease.

Direct immunofluorescence could not be performed due to the unavailability in our setup.

The patient was started on oral naltrexone 1.5 mg once daily for 2 weeks, followed by 1.5 mg twice daily for 2 weeks, along with topical tacrolimus 0.1% ointment and emollients.

## 4 Follow‐Up and Outcome

At one‐month follow‐up, the patient showed marked clinical improvement. The patient is continued on the same treatment and planned for follow‐up in 1 month.

## 5 Discussion

Hailey–Hailey disease is a chronic, relapsing genodermatosis caused by mutations in ATP2C1 that impair keratinocyte adhesion. It typically presents as recurrent vesicles, erosions, fissures, and macerated plaques mainly in intertriginous areas such as the axillae, groins, neck, inframammary folds, and other friction‐prone sites [1, 2]. Our patient exhibited these classic features on the nape of the neck and axillae, with recurrences in summer and worsening with sweating and friction, closely aligning with the clinical pattern noted in recent literature [1, 2].

However, the diagnostic challenge in our case was due to the additional papular and follicular morphology, with reticulate‐to‐patchy pigmentation over the groin, lower abdominal folds, and popliteal fossae, as well as marked post‐inflammatory pigmentation. This presentation differed from the more frequently described erosive and macerated plaques typically seen in HHD. A case report from Nepal by Thapa et al. [7] described a patient with pruritic hyperkeratotic papules and plaques affecting the vulva, perianal region, and inner thighs, demonstrating that Hailey–Hailey disease may present with papular or hyperkeratotic morphology rather than solely classical erosive plaques. In contrast, other clinicopathological reports have predominantly described the classical presentation of recurrent vesicular and erosive flexural lesions with a “dilapidated brick wall” pattern of acantholysis [6]. Thus, the prominent follicular‐papular morphology and pigmentation observed in our patient represent a less commonly emphasized variant.

The primary differential diagnoses considered were Darier's disease, transient acantholytic dermatosis (Grover's disease), and Dowling–Degos disease. Grover's disease can show focal acantholysis on histology and can mimic Hailey–Hailey disease microscopically; however, it typically presents as pruritic papules or papulovesicular lesions predominantly on the trunk in older patients and tends to be transient [5]. Conversely, our patient's persistent, relapsing course, especially in flexural areas, along with summer recurrences and worsening with sweat and friction, pointed toward Hailey–Hailey disease. Darier's disease typically presents with keratotic papules in seborrheic regions, along with nail abnormalities and acantholytic dyskeratosis—features not seen in our case [3]. Dowling–Degos disease was also considered, given the reticulate pigmentation and flexural involvement, but the absence of characteristic comedo‐like lesions and pitted scars made this diagnosis less likely [4].

Histopathology was crucial in establishing the diagnosis. A biopsy from the nape of the neck showed suprabasal acantholysis with keratinocyte separation, creating the characteristic dilapidated brick‐wall pattern consistent with Hailey–Hailey disease [1, 6]. This finding helped exclude Dowling–Degos disease, which does not exhibit acantholysis and instead shows elongation and branching of rete ridges with increased basal pigmentation [4]. The absence of prominent dyskeratosis, such as corps ronds and grains, further argued against a diagnosis of Darier's disease [3]. Additionally, the chronic, recurrent nature of the condition with primarily flexural involvement made transient acantholytic dermatosis less likely [5].

A positive family history was absent in our patient. Although Hailey–Hailey disease is typically inherited in an autosomal dominant manner, a positive family history may be absent. It may reflect variable penetrance, unrecognized mild disease, or de novo mutations [1, 2]. A similar finding was reported by Thapa et al. [7] despite histological confirmation. Therefore, lack of family history should not preclude the diagnosis when clinical and histopathological features are supportive.

Management of Hailey–Hailey disease includes avoidance of heat, friction, and sweating, as well as topical corticosteroids and antimicrobials. Various systemic and procedural therapies have been used in refractory cases with variable success [1, 2]. Low‐dose naltrexone has emerged as a therapeutic option for difficult cases of HHD, with case series and reports demonstrating clinical improvement at doses typically between 1.5 mg and 4.5 mg daily [8, 9]. McBride et al. documented improvement in a patient with longstanding HHD following low‐dose naltrexone, whereas Lim et al. reported better outcomes for recurrent inguinal HHD when combining low‐dose naltrexone with oral magnesium chloride [9, 10]. Although our patient is at an early stage of treatment and further follow‐up is needed, this regimen is supported by recent evidence.

## 6 Conclusion

This case highlights the diagnostic challenge posed by atypical morphological variants of Hailey–Hailey disease, particularly when lesions exhibit papular and follicular features with prominent post‐inflammatory pigmentation. It underscores the importance of thorough clinicopathological correlation in differentiating it from other acantholytic dermatoses, such as transient acantholytic dermatosis.

## Author Contributions

**Sandesh Shah:** conceptualization, writing – original draft, methodology, project administration, writing – review and editing. **Joshana Shrestha:** conceptualization, writing – original draft, writing – review and editing, methodology, project administration, resources. **Rabin Baniya:** writing – review and editing, resources, methodology. **Mohan Bhusal:** writing – review and editing, methodology, resources. **Krishangi Pradhan:** writing – review and editing. **Radhika Maharjan:** writing – review and editing.

## Funding

The authors have nothing to report.

## Consent

Written informed consent was obtained from the patient in accordance with the journal's patient consent policy. I will retain the original written consent form and provide it to the Publisher if requested.

## Conflicts of Interest

The authors declare no conflicts of interest.

## Data Availability

The authors have nothing to report.
